# Complexity of matrix phenotypes

**DOI:** 10.1016/j.mbplus.2020.100038

**Published:** 2020-05-28

**Authors:** Renato V. Iozzo, Achilleas D. Theocharis, Thomas Neill, Nikos K. Karamanos

**Affiliations:** a Department of Pathology, Anatomy and Cell Biology and the Cancer Cell Biology and Signaling Program, Kimmel Cancer Center, Thomas Jefferson University, Philadelphia, PA, United States of America; b Biochemistry, Biochemical Analysis and Matrix Pathobiology Research Group, Laboratory of Biochemistry, Department of Chemistry, University of Patras, Patras, Greece

**Keywords:** ADAM, a disintegrin and metalloproteinases, AGE, advanced glycation end products, DDR1, discoidin domain receptor 1, ECM, extracellular matrix, EGF, epidermal growth factor, EGFR, epidermal growth factor receptor, EMILIN1, elastin microfibril interfacer 1, EMILIN2, elastin microfibril interfacer 2, EMT, epithelial-mesenchymal transition, ERα, estrogen receptor α, ERβ, estrogen receptor β, GBM, glioblastoma, HA, hyaluronan, HAS2, hyaluronan synthase 2, HAS2-AS1, HAS2 antisense 1, HB-EGF, heparin binding EGF, HMGA2, high-mobility group AT-Hook 2, IBC, inflammatory breast cancer, IGF-IR, insulin growth factor I receptor, IR-A, insulin receptor A, LEKTI, lympho-epithelial Kazal-type inhibitor, LOX, lysyl oxidases, LTBP, latent TGFβ-binding proteins, MAGP, microfibril-associated glycoproteins, MET, mesenchymal-epithelial transition, miR, microRNA, MMP, matrix metalloproteinases, OB, osteoblast, OI, osteogenesis imperfecta, PARs, protease activated receptors, PG, proteoglycans, PLL, poly-l-lysine, ROS, reactive oxygen species, RTK, receptor tyrosine kinase, SLRP, small leucine rich proteoglycans, SSR, solar-simulated radiation, TGFβ, transforming growth factor β, TNT, tunneling nanotubes, tPA, tissue-type plasminogen activator, uPA, urokinase-type plasminogen activator, UVR, ultraviolet radiation, VEGF, vascular endothelial growth factor, Proteoglycans, Angiogenesis, Collagen, Cancer, Methodologies

## Abstract

The extracellular matrix is engaged in an ever-evolving and elegant ballet of dynamic reciprocity that directly and bi-directionally regulates cell behavior. Homeostatic and pathophysiological changes in cell-matrix signaling cascades manifest as complex matrix phenotypes. Indeed, the extracellular matrix can be implicated in virtually every known human disease, thus, making it the most critical and dynamic “organ” in the human body. The overall goal of this *Special Issue* is to provide an accurate and inclusive functional definition that addresses the inherent complexity of matrix phenotypes. This goal is summarily achieved via a corpus of expertly written articles, reviews and original research, focused at answering this question empirically and fundamentally via *state-of-the-art* methods and research strategies.

## Highlights

• ECM is the most important “organ” of the body; virtually all pathologies emanate from aberrant cell-matrix interactions
• Matrix phenotypes manifest from ECM molecules and signaling into the cell nucleus as a form of “*dynamic reciprocity*”
• This review focuses on the complexity of matrix phenotypes as diverse as the extracellular matrix itself
• State-of-the-art methods, from ex vivo to next generation “*omics*” assays, bring intricate matrix phenotypes into focus
• Deciphering the complexity of matrix phenotypes will uncover novel therapeutics and biomarkers to diagnose and treat disease

## Introduction

Defining a concept as broad as a “matrix phenotype” that accurately encompasses the remarkable diversity of our field is challenging. This is due to the intrinsic complexity, yet elegance, of the extracellular matrix (ECM). Indeed, the ECM can be considered among the most complex organs in the human body. Nearly 40 years ago, Mina Bissell and colleagues began to unravel its complexity [1]. In these seminal works, an enduring model, known as “*dynamic reciprocity*” [2,3] was conceived to mechanistically elucidate how matrix signals are transduced and integrated to affect nuclear gene expression programs that ultimately govern hierarchical phenotypes.

At the core of this insightful model are direct signaling cascades responsible for remodeling the cellular inner structure, resulting in alterations to afferent and efferent signaling systems. This occurs at the cell surface and involves integrins and other transmembrane cell surface receptors that interact with matrix molecules, such as proteoglycan receptors [4,5] via their dichotomous interactions with the ECM and internal cytoskeletal components. These coordinated and often synergistic interactions are transduced to the nuclear matrix for precise spatiotemporal control over gene expression programs. Extending the model further, this functional paradigm predicts that nearly all human pathophysiologies may have an underlying matrix phenotype [[6], [7], [8], [9], [10], [11], [12], [13], [14], [15], [16], [17]], thus reflecting the pervasiveness and complexity of cell-matrix interactions. Therefore, it is the goal of this compendium to convey the latest findings and methodologies used to decode matrix signaling and their myriad interactions that fundamentally provides a representative definition of the evolving complexity of matrix phenotypes.

## Extracellular matrix remodeling induced by radiation

The ECM is a macromolecular network surrounding cells to form tissues and organs with an intrinsically unique molecular and structural signature for their specific functional properties. Key protagonists are proteoglycans; they orchestrate receptor cross-talk during various pathologies [[18], [19], [20], [21], [22], [23], [24], [25], [26], [27], [28]] and function as key structural and ECM-modifying elements [[29], [30], [31]]. ECM molecules undergo physiological remodeling and exhibit long half-lives that accumulate molecular damage due to aging and chronic diseases [15,32]. As an example, skin is the largest organ of human body that photoages when exposed to ultraviolet radiation (UVR). In response to UVR, the elastic fiber network within the skin ECM undergoes an accelerated loss of fibrillin microfibrils. Composed of fibrillins, fibulins, microfibril-associated glycoproteins (MAGPs), and elastin microfibril interfacer 1 (EMILIN-1), these fibrillin microfibrils regulate elastogenesis and are made up of several modular repeats including calcium-binding epidermal growth factor (EGF)-like repeats, transforming growth factor β (TGFβ)-binding like domains as well as hybrid domains that exhibit homology to both domains. In addition, fibrillin and fibrillin-associated proteins including fibronectin, fibulins, and LTBPs contain high amounts of UVR-sensitive amino acid residues (disulfide-bonded cysteine, tryptophan, and tyrosine) that act as chromophores [33]. Separately, fibrillins not only make up a key component of fibrillin microfibrils but also bind to numerous other ECM components such as fibulins, MAGPs, perlecan and hyalectans, latent TGFβ-binding proteins (LTBPs), fibronectin, growth factors, enzymes, and cell surface receptors via their modular domains. They maintain the structural integrity of ECM, anchor the interstitial matrix to the basement membrane, and participate in cell-matrix interactions [34,35]. Collagen type VI microfibrils are also important for connecting the basement membrane to the stroma via collagen IV, perlecan, and interstitial matrix molecules including collagen I/III heterotypic fibrils, fibronectin, decorin, and biglycan [[35], [36], [37]].

**Fig. 1:**
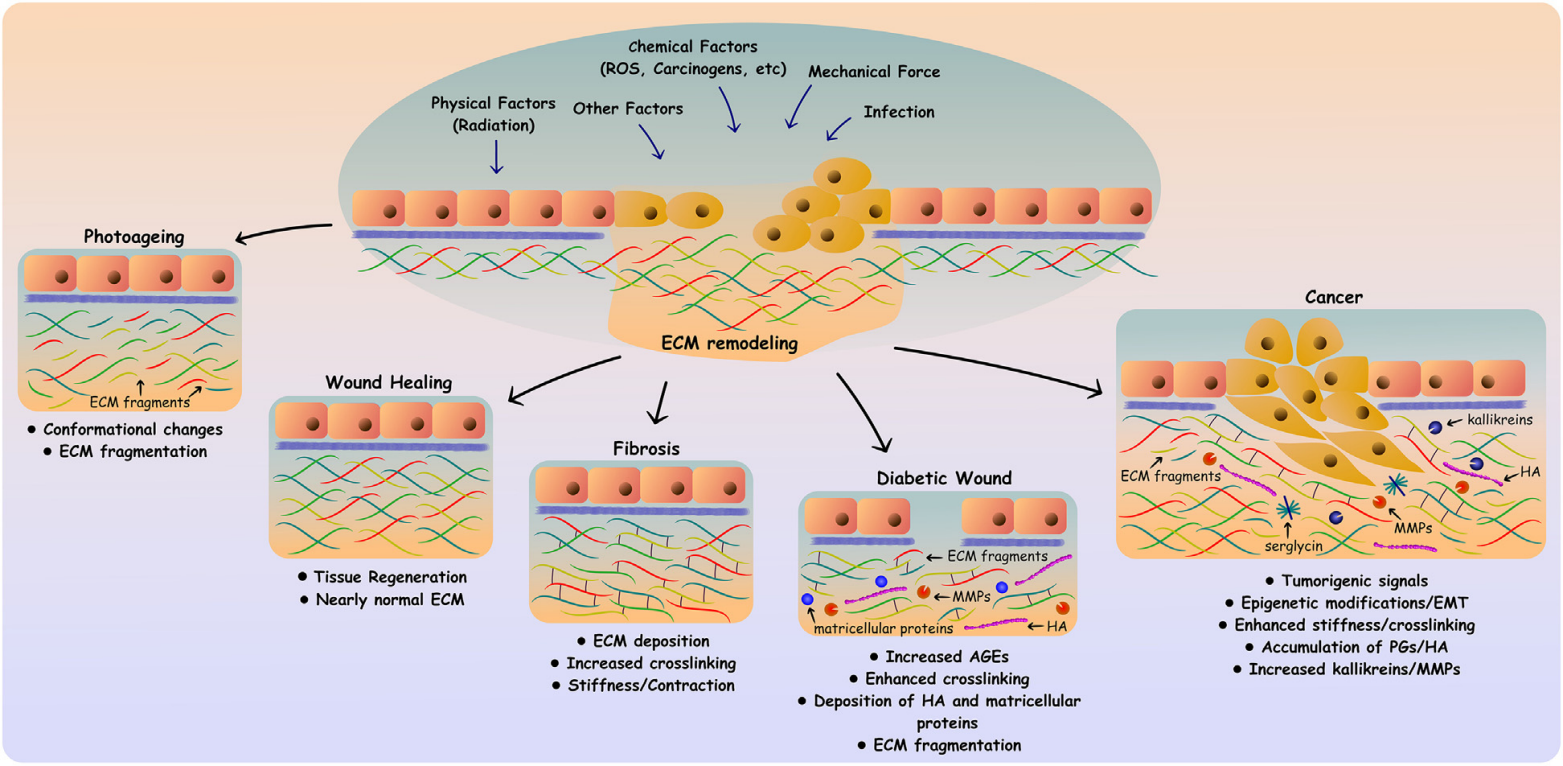
Schematic representation of the complexity of matrix phenotypes. The ECM is a well-organized three-dimensional and interconnected lattice of tissue and context-specific macromolecules and enzymes for critical post-translational modifications. These macromolecules create a dynamic and functional network that bi-directionally regulates cell behavior and manifests as an array of matrix phenotypes. A multitude of physical, chemical, and mechanosensitive cues, as well as bacterial infections, can result in tissue damage often associated with extensive ECM remodeling. Please refer to the text for the specific examples diagrammed here that occur during homeostasis and pathological states.

Exposure to UVR by broadband UVB and solar-simulated radiation (SSR) causes significant ultrastructural changes exclusively in fibrillin microfibrils as detected by the enhanced periodicity and central bead height of the irradiated microfibrils in vivo and in vitro [38]. UVR remodels the tertiary and quaternary structure of fibrillin microfibrils making them more susceptible to proteolysis as measured by an increase in liberated peptides as measured via LC-MS/MS analyses following exposure to elastase (Fig. 1). Notably, fibrillin-1 and collagen VI exhibit UVR-specific regional foci of elastase susceptibility. Although collagen VI is mostly resistant to elastase after UVR-exposure, UVR-induced regional foci of increased elastase susceptibility do arise post-irradiation, suggesting that certain molecular conformations exist for protease sensitivity. Hence, LC-MS/MS mapping of liberated peptides is a powerful and sensitive tool for identifying and characterizing early post-translational damage of ECM molecules caused by various insults, including reactive oxygen species (ROS) and UVR.

## Emerging tools that reveal and treat matrix phenotypes

Innovative, next-generation modalities such as those discussed above are rapidly emerging to address a plethora of scenarios. These methods also take the form of classical ex vivo assays such as the aortic ring assay to functionally elucidate the mechanisms of ECM signaling. Kapoor et al. elegantly present a simplified methodology of performing aortic ring assays to examine the role of matrix signaling in the context of ex vivo angiogenesis. This entails modifying the technique by embedding the rings between two layers of collagen type I with vascular endothelial cell media. Thus, it is a true 3D system that can allow investigating in real time the effects of biomolecules and pharmacological agents that affect sprouting. The article then describes how to obtain high-resolution confocal microscopy images and a novel method to extract the growing vessels for biochemical analyses. This method could be instrumental in studying the role of biochemical, pharmacological and protein factors such endorepellin [[39], [40], [41], [42], [43], [44], [45], [46]], decorin [[47], [48], [49], [50], [51], [52], [53]], or biglycan [54] in modulating angiogenesis and autophagy.

The promise of therapeutically targeting components of the matrix with powerful gene therapy approaches is elegantly examined in a report by Maruelli et al. using osteogenesis imperfecta (OI) as a congenital disease model in which dominant mutations in the genes encoding the α chains of collagen type I (*COL1A1* and *COL1A2*) manifests as a rare brittle bone disease [55]. In this report, authors used a gene therapy approach to silence bone-specific *COL1A2*. Importantly, individuals that lack the α2(I) chain do not show a brittle bone phenotype. Thus, the authors engineered three different siRNA oligonucleotides that specifically targeted murine *Col1a2*. Efficiency and specificity were validated in vitro and in vivo, with significant silencing achieved in both systems. Importantly, biphasic calcium phosphate implants loaded with murine mesenchymal stem cells, transplanted intramuscularly into nude mice and injected with *Col1a2*-siRNA-3554 resulted in a newly formed collagen matrix. These data provide the exciting possibility that specifically silencing *Col1a2* via siRNA delivery may represent a viable therapeutic approach for OI. Collectively, the “*-omic*” methods of LC-MS/MS combined with updated functional assays, such as ex vivo aortic rings, and applied gene therapy approaches provide powerful tools to reveal and ameliorate disease phenotypes in the matrix.

## Matrix phenotypes associated with wound healing

A multitude of other external factors including chemical compounds, mechanical forces, and bacterial infections, among others, can cause tissue damage and wounds (Fig. 1). The ECM routinely undergoes extensive remodeling to support all essential cellular and structural processes during wound healing [15,35,56]. The final outcome is tissue regeneration that nearly restores previous tissue structure and functions or fibrosis with modified tissue properties (Fig. 1). Under specific circumstances, such as in diabetes, impaired wound healing may occur that leads to the development of ulcerated wounds. The development of diabetic wounds is associated with unique ECM phenotypes and disease complications such as diabetic foot ulcer. In this *Special Issue*, Huang and Kyriakides review the alterations of ECM molecules germane for diabetic wound healing. Hyperglycemia promotes glycation-induced modifications of ECM proteins and the formation of advanced glycation end products (AGEs) and compromised ECM function (Fig. 1). For example, the formation of glucosepane, the most abundant and clinically relevant AGE crosslink in type I collagen-rich tissues, is associated with changes to the structural and physical properties of collagen fibrils and many complications of diabetes [57]. Furthermore, severe changes in the production of matrix molecules (Fig. 1) occur in diabetic wounds such as reduced collagen deposition, increased levels of matricellular proteins, hyaluronan (HA), and altered proteoglycan (PG) expression.

Also in this *Special Issue*, Colineau et al. report on a novel aspect of matrix phenotypes that arises at the intersection of ECM components and infectious agents. They discovered that several small leucine rich proteoglycan (SLRP) family members, including biglycan, decorin, fibromodulin and osteoadherin, contain inherent anti-microbial properties that can eradicate infection. The microbial agent of interest is *Streptococcus pyogenes* (*S. pyogenes*) that is responsible for a spectrum of conditions, ranging from mild clinical syndromes to fatal outcomes. During the most severe infections, *S. pyogenes* interacts with members of the SLRP family. This aligns with a multitude of immunomodulatory functions ascribed to the SLRP family [20,31,[58], [59], [60], [61], [62]], particularly for the class I SLRPs, decorin and biglycan [[63], [64], [65], [66], [67], [68], [69]]. Indeed, soluble biglycan is considered a biomarker for inflammatory kidney disease [[70], [71], [72], [73]]. Infection of human monocytes with *S. pyogenes* results in decorin expression, akin to previous findings [66]. Importantly, multiple genetically distinct and clinically relevant strains of *S. pyogenes* physically interact with several SLRPs, resulting in decreased survival. Surprisingly, the ability of decorin and biglycan to eliminate *S. pyogenes* was independent of canonical decorin and/or biglycan binding to TLR2/4. Instead, these SLRPs increased deposition of complement complexes from human serum and its activator, C1q, onto the bacterial surface of *S. pyogenes*. This resulted in efficient microbial elimination. Collectively, this innovative study further builds upon the functional repertoire of SLRPs as eradicators of dangerous bacterial infections.

The kallikreins represent a family of 15 serine proteases with chymotrypsin or trypsin-like activities. Initially, the proteolytic cascade of kallikreins in the skin is activated by auto-activation of kallikrein 5 that, in turn, cleaves and converts kallikrein 7, 14, and 6 to their active forms. These activated kallikreins can further activate other family members and cleave ECM components for remodeling. Their activity is regulated by binding endogenous serine protease inhibitors such as lympho-epithelial Kazal-type inhibitors (LEKTI and LEKTI2). The interaction of kallikreins with LEKTI is pH-dependent, and active kallikreins are released by these complexes in low pH conditions [74]. Nauroy and Nyström attractively review the expression and functions of kallikreins in skin, cancer progression and EMT (Fig. 1). Several kallikreins are differentially expressed in various layers of epidermis and they participate in skin homeostasis and repair as well as in the development of skin diseases. Kallikreins participate in skin desquamation, the physiological shedding of the outermost layer of epidermis. This is mediated via their ability to cleave transmembrane proteins such as desmoglein 1, desmocollin 1 and the ECM protein corneodesmosin. They are involved in the formation of corneo-desmosomes, specialized junctions involved in the adhesion of differentiated keratinocytes (corneocytes) in the outermost layer of the epidermis. Kallikreins 6/7 can either directly, or via activation of MMPs and metalloproteinases (ADAMs), cleave other junctional proteins, such as E-cadherin, to promote proliferation and migration [74]. Kallikreins are released into the wound bed from damaged epidermis, are activated at low pH, as found in the wound microenvironment, and aid in the wound healing process. Kallikrein 3 and 5 convert plasminogen into plasmin to remodel fibrin clots. In turn, plasmin activates other kallikreins to enhance their functions, in a positive feedback loop. Kallikreins degrade several ECM components including collagen I, collagen III, fibronectin, and vitronectin that participate in matrix remodeling and ultimately the deposition of a provisional matrix essential for wound healing and re-epithelialization [74,75].

The kallikreins modulate intracellular signaling cascades via cell surface receptor engagement. Indeed, kallikreins activate EGF receptor (EGFR) signaling to evoke keratinocyte migration and proliferation [74]. Kallikreins can also cleave protease activated receptors (PARs), leading to PKC activation and intracellular signal propagation, which promotes ADAMs/MMPs. Activated MMPs shed cell surface heparin binding EGF (HB-EGF) and consequently activate EGFR. Alternatively, kallikreins may directly activate cell-surface MMPs/ADAMs or directly shed HB-EGF to activate EGFR signaling [74].

### Malignant matrix phenotypes and prognostic biomarkers for tumorigenesis

Epithelial–mesenchymal transition (EMT) is a fundamental, physiologic mechanism during development. EMT can also be activated during wound healing, fibrosis, and cancer. EMT is associated with loss of cell–cell adhesions and the de-diffentiation of apico-basal polarity characteristic of epithelial cells. Epithelial cells are transformed into mesenchymal, spindle-like cells which possess increased migratory, invasive, and metastatic properties, a phenotypic modulation prevalent in malignant transformation. EMT is closely related to cytoskeletal re-arrangement, and linked to modified populations of cell surface receptors, transcription factors, growth factors, and cytokines. EMT reprogramming is further associated with altered cell signaling and epigenetic modifications [76]. EMT provides cancer cells with resistance to oncogene-induced senescence and chemo- or radio-therapeutic regimes, thus contributing to the generation of cancer stem cells that drive the long-term maintenance and metastatic dissemination of the disease. Mesenchymal-type cancer cells also participate in the remodeling of ECM either directly or indirectly by regulating cell behavior of stromal cells to facilitate tumorigenesis. Notably, EMT is transient and mesenchymal cancer cells can undergo the inverse to re-acquire less aggressive epithelial phenotypes via mesenchymal-epithelial transition (MET). Malignancies, among other pathologies, are considered ECM-driven diseases, and indeed, the newly-formed stromal microenvironment is critical for disease progression [13]. Aberrantly remodeled ECM regulates EMT/MET in cancer cells, thereby controlling cancer cell plasticity and functions [76] (Fig. 1) and manifesting malignant matrix phenotypes. Adding to the repository of ECM constituents that have gained increased attention for their involvement in EMT, is collagen XV and its wide array of pleiotropic effects. For the first time, an elegant and comprehensive review by In this *Special Issue*, Bretaud et al. compiled the current state of knowledge on this intriguing, and often cryptic, collagen/proteoglycan hybrid.

Recently, a pan-cancer study of matrisome gene expression in 10,487 patients across 32 different tumor types, supplemented with transcription factors and driver mutations has been published [77]. Specific transcription factors and master regulators govern a unique matrisome for each cancer. These data provide an extensive transcriptional portrait and suggests the importance of designing specific cancer matrisome-targeted approaches as future therapies [77]. Liot et al. in this *Special Issue* demonstrate the significance of tenascin-X mRNA expression in 13 types of cancer. This was done by analyzing 90 GSE datasets from the Gene Expression Omnibus database and using the UALCAN interactive web-portal to perform in-depth analyses of TCGA gene expression data [78]. Down-regulation of tenascin-X in most tumors, except gliomas, represents a unique ECM signature associated with poor prognosis in breast and lung cancer. This is congruent with findings that tenascin-X is differentially regulated compared to other tenascins, such as tenascin-C, in cancer [79]. Tenascin-C induces EMT to enhance migration and survival via TGF-β for lung metastasis [80].

Deciphering the expression profiles of ECM components in cancer is becoming a vital source of novel diagnostic and prognostic biomarkers. This is aptly demonstrated by a series of papers within this *Special Issue*. Andreuzzi et al. evaluate the role of EMILIN2 in gastric cancer. This human malignancy lags in available options for targeted therapy as compared to the progress made with other solid tumors. It is well established that main member of this family, EMILIN1, interacts with the β1-containing integrins [81] and the gC1q domain [82], and abrogation of EMILIN1-β1 integrin interactions promotes colon carcinoma progression [83]. Notably, the expression of EMILIN2 is consistently and markedly suppressed in gastric cancer, but high levels of this glycoprotein are linked to abnormal vascular density. The authors further discovered that EMILIN2 has a dual functionality in gastric cancer. EMILIN2 suppresses gastric carcinoma cell proliferation via apoptotic induction; however, it also evoked synthesis of cytokines, including IL-8 that promotes inflammation and angiogenesis.

In a study by El-Nadi et al., a strongly positive correlation of heparanase was discovered in cases of inflammatory breast cancer (IBC) when compared with non-IBC cases. Using immunostaining and assigning a signal intensity score, heparanase correlated with infiltrating CD163+ M2 tumor-associated macrophages (TAMs) specifically within IBC. Buraschi et al. found that discoidin domain receptor 1 (DDR1), a critical collagen receptor, functionally interacts with the IGF-I system in bladder cancer. This physical interaction results in cross-talk among DDR1, IGF-IR, and insulin receptor A (IR-A) for bladder cancer progression. DDR1 was expressed in invasive and metastatic uroepithelial cells but devoid in non-invasive cells. Stimulation with IGF-I, IGF-II, or insulin resulted in DDR1 activation and binding to IGF-IR and IR-A. Functionally, these interactions led to F-actin dynamics via Pyk2 and non-muscle myosin IIA in a ligand-dependent manner. This signaling cascade mechanistically links the IGF-IR and IR-A to cytoskeleton remodeling for increased bladder cancer cell motility and invasion. Therefore, DDR1 may represent a novel biomarker for bladder cancer.

Hyaluronan (HA) is highly-expressed within the tumor stroma and in other pathological states. HA fragments of differing molecular weights may be generated during these processes and some may exhibit pro-inflammatory activity and serve as a potent trigger for tumorigenesis via cell surface receptors [[84], [85], [86], [87], [88], [89]] (Fig. 1). Interestingly, it has been shown that HA synthase 2 (HAS2) is required for efficient TGF-β-mediated EMT [90]. TGFβ induces expression of the natural antisense transcript of HAS2 (HAS2-AS1) and high-mobility group AT-hook 2 (HMGA2) via Smad-dependent and independent pathways [91]. HAS2-AS1 induces *HAS2* transcription via protein O-GlcNAcylation [92]. HAS2-AS1 is key in TGF-β- and HAS2-induced breast cancer EMT and cancer stemness as increased HAS2 secretes HA that promotes EMT via CD44 [91]. Several therapeutic approaches, including drugs affecting HA synthesis and peptide mimetics that directly bind HA or its receptors (CD44 and RHAMM), are proposed for treating HA-related pathologies [93,94].

Karalis et al. in this *Special Issue* demonstrate that treatment of triple negative breast cancer cells with salicylate (aspirin), a commonly used anti-inflammatory agent, affects the oncogenic HA network and cancer cell proliferation and migration due to decreased cyclin D expression and re-distribution of CD44 and actin cytoskeleton, respectively. Regarding the HA network, treatment with salicylate results in AMPK activation, decreased HA production associated with decreased HAS2 levels and concurrent induction of hyaluronidase 2 in breast cancer cells. Notably, HAS2 can be reduced by autophagic degradation of the enzyme, thereby providing a novel way of counteracting pro-inflammatory and pro-angiogenic HA [95].

Intracellular PGs, such as serglycin, are constitutively secreted into the ECM by a large array of cells and play a central role in oncogenesis by aberrantly activating signaling cascades resulting in aggressive phenotypes [96,97] (Fig. 1). Serglycin is highly expressed in aggressive cancer cells and its post-translational modification (glycanation) and its efficient secretion are required to induce growth, migration, and invasion [98]. Serglycin induces multiple pro-tumorigenic signaling pathways evoking EMT and cancer stem cell properties in several cancer cell types [[99], [100], [101]]. Mechanistically, serglycin interacts with CD44 and/or as-of-yet unknown cell surface receptors to activate intracellular signaling [[99], [100], [101]] and the production of pro-inflammatory mediators and ECM remodeling enzymes [99,102]. In this *Special Issue*, Manou et al. demonstrate that serglycin is essential for the heterotopic in vivo growth of glioblastoma (GBM) in mice. Serglycin suppression evokes differentiation of GBM to less aggressive astrocytoma that exhibits reduced growth, migratory, invasive, and stemness properties. Phenotypic differentiation of GBM to astrocytoma is associated with reduced secretion of pro-inflammatory cytokines such as IL-6 and IL-8 as well as decreased expression of proteolytic enzymes including MMPs and urokinase-type plasminogen activator (uPA). Serglycin-suppressed cells also present repressed activation of ERK1/2, p38, SRC, and STAT-3 that, together with PI3K and IL-8/CXCR2 signaling, controls GBM aggressiveness. Thus, this intriguing proteoglycan, once thought to be a specific intracellular proteoglycan within mast cell granules, can play a role in several malignancies.

### Estrogen receptors control breast cancer cell phenotype and matrix remodeling

Estrogen receptor α (ERα) and estrogen receptor β (ERβ) are among the nuclear receptors that act as ligand-activated transcription factors that regulate EMT and ECM remodeling in breast cancer [[103], [104], [105]]. Suppression of ERα in luminal MCF-7 breast cancer cells induces EMT and functional properties associated with induced MMPs and uPA/tissue-type plasminogen activator (tPA) concomitant with enhanced EGFR-ERK1/2 signaling [103]. Interestingly, it has been shown that IGF-IR and ERα cooperate to inhibit the aggressive phenotype by regulating proteolytic enzymes and cell surface receptors, including syndecans and integrins [106]. On the other hand, aggressive triple negative MDA-MB-231 cells, which express ERβ, exhibit phenotypic and functional alterations consistent with MET upon ERβ suppression. ERβ-suppressed breast cancer cells have a less aggressive phenotype characterized by reduced expression of proteolytic enzymes, PGs and EGFR/IGF-IR and inhibited JAK/STAT signaling [104].

Suppressing ERβ in MDA-MB-231 cells results in epigenetic alterations regarding specific microRNAs (miR), including miR-10b, miR-200b, and miR-145 [107]. OncomiRs emerged not only as critical phenotypic regulators, but also as key players in ECM remodeling in physiological and pathophysiological processes [56,108] (Fig. 1). ECM remodeling by miRs affects matrix molecule and proteolytic enzyme expression [109]. The levels of miR-10b are decreased in ERβ-suppressed MDA-MB-231 cells leading to a less aggressive phenotype affecting EMT and proteolytic potential. Conversely, up-regulation of miR-145 in ERβ-suppressed MDA-MB-231 cells promotes a similar less invasive phenotype [107]. Piperigkou et al. show that increased miR-200b occurs in less aggressive breast cancer cells and is associated with longer overall patient survival. Overexpression of miR-200b restrains EMT and invasive potential and is dependent on ER status. miR-200b evokes MET and reduces MMP expression and functional properties only in mesenchymal type ERβ-positive MDA-MB-231 cells and not in luminal, ERα-positive MCF-7 cells. Thus cross-talks between nuclear receptors/transcription factors and microRNAs can significantly affect the malignant phenotype in mammary carcinomas.

In this *Special Issue*, Franchi et al. demonstrate that highly aggressive MDA-MB-231 and less aggressive ERβ-suppressed MDA-MB-231 cells form tunneling nanotubes (TNTs), which allow direct communication between cells, and very long filopodia when cultured on ECM in the presence of estradiol. Interestingly, TNTs are often associated with adhering exosomes and microvesicles. Estradiol affects ECM molecule expression, mainly in cells expressing ERβ. Further studies are needed to elucidate the importance of TNTs in cell-cell and cell-matrix interactions in cancer.

### A microcosm of matrix phenotypes: role of ECM in intervertebral disc degeneration

A prime example of how ECM underlies pathophysiological states is found in the intervertebral disc. In a comprehensive study by Ohnishi et al., the relative levels of aggregating proteoglycans as well as fibrillar collagens were found to exert a major role in the biomechanical functions of the disc that lead to fibrosis. Profiling these constituents revealed an altered ECM signature that signifies various complex matrix phenotypes that manifest clinically as disc degeneration. Key roles of these ECM structures are to impart spinal column flexibility and to absorb mechanical stresses, which is directly conferred by the population of ECM molecules. Due to their relatively high concentration of sulfated GAG chains, aggrecan and versican are able to form large aggregates, along with HA, to sequester water molecules within the disc and provide resiliency and flexibility. The role of syndecan-4 in intervertebral discs is currently a matter of debate [110]. Mechanistically, the RNA binding protein HuR has been found to regulate a subset of matrix genes independently of HIF-1α in the nucleus pulposus cells [111]. However, multiple factors can impair the homeostatic functions of aggregan, which can have egregious effects on the function of the disc. These adverse events include increased degradation of the aggrecan protein core via MMPs and aggrecanase in response to pro-inflammatory cytokines, growth factors [112] and immune cell activation. Loss of TonEBP, a critical disc ECM regulator [113], accelerates disc degeneracy via increased matrix remodeling and changes in the expression of genes encoding pro-inflammatory modulators [114]. Errors in the form of single nucleotide polymorphisms affect the activity of CS-synthesizing enzymes as well as the actual attachment sites to aggrecan [115]. Moreover, it is not just the aggregating proteoglycans that are affected as many other ECM molecules, such as CCN2 [116], are dysregulated during disc degeneration and herniation as demonstrated via elegant genetic approaches [117]. In the initial stages of disc degeneration, there is a significant increase in apoptosis and the loss of cell phenotype. This phase is typically followed by a wave of fibrotic matrix deposition that includes several collagens, fibronectins, and SLRPs. In aging discs, the senescence-associated secretory phenotype is also connected to disc matrix homeostasis as loss of the CDKI, p16^Ink4a^, impairs normal ECM without altering senescence [118]. Collectively, these observations reinforce the concept of ECM complexity and the emergence of matrix phenotypes in intervertebral disc degeneration.

## Conclusions

We hope that this compilation of articles would serve as a general exposé on the complexity of matrix phenotypes. Encompassing human pathologies as diverse as skin diseases, bacterial infections, cardiovascular maladies, fibrosis, osteogenesis imperfect, and tumorigenesis, the unique ECM profiles have a pivotal role in each. The “dynamic reciprocity” underlying each disease does, indeed, have a specific rhythm that manifests in an inimitable matrix phenotype. Understanding the intricate nuances of each can lead to more advanced and precise therapeutic modalities, applications, and matrix-centric biomarkers. This knowledge is significantly deepened by the articles assembled in this first *Special Issue* that collectively define the ever-evolving complexity of matrix phenotypes.

## Declaration of competing interest

The authors declare no conflicts of interest.
